# Imprisonment for South Ethiopian people living with HIV presents a double health burden: lived experiences of prisoners

**DOI:** 10.1186/s12913-024-10587-y

**Published:** 2024-01-22

**Authors:** Terefe Gone Fuge, George Tsourtos, Emma R Miller

**Affiliations:** 1 School of Public Health, Wachemo University, Hossana, Ethiopia; 2 Torrens University, Adelaide, Australia; 3 The Stretton Institute, Stretton Health Equity, The University of Adelaide, Adelaide, Australia

**Keywords:** Inmates living with HIV, Care use, Barriers and facilitators, South Ethiopia

## Abstract

**Background:**

Optimal adherence is crucial for ensuring both therapeutic and preventative benefits of antiretroviral therapy (ART). Sub-optimal adherence is common in prisoners and little information is available about its predisposing circumstances in resource-limited settings. We explored lived experiences of inmates living with HIV (ILWH) and experiential accounts of service providers in South Ethiopia to identify barriers to and facilitators of HIV care use in the prison context.

**Methods:**

We conducted qualitative in-depth interviewing with eleven ILWH and eleven service providers. Audio recorded interview data were transcribed verbatim in Amharic language, translated into English and coded based on emerging concepts. We employed a descriptive phenomenological approach to abstract meaning attributed to the prisoners’ lived experiences in relation to HIV care use and service providers’ experiential account regarding care provision as presented to our consciousness.

**Findings:**

Several concepts emerged as barriers to HIV care use amongst ILWH in South Ethiopia including: limited access to standard care, insufficient health staff support, uncooperative security system, loss of patient privacy, a lack of status disclosure due to social stigma, and food supply insufficiency. In addition to a unique opportunity offered by an imprisonment for some ILWH to refrain from health damaging behaviours, the presence of social support in the prison system facilitated care use.

**Conclusions:**

This study identified important structural and social contexts that can both hinder and enhance HIV care use amongst ILWH in South Ethiopia. Given the disproportionate burden of HIV in prisoners and the potential of transmission to others during and after incarceration, development of contextually-responsive strategies is required to address the barriers and to also strengthen the enablers.

## Background

Optimum adherence to antiretroviral therapy (ART) (i.e., taking ≥ 95% of prescribed medication) is essential to achieve viral suppression, increase survival rates in people living with HIV (PLWH), and to prevent onward transmission [1–3]. Sub-optimal adherence can also cause drug-resistance which leads to increased use of costly second line drugs [4–6]. Prisoners are among key populations that bear a disproportionate burden of HIV epidemic and have a greater potential of transmitting to others during and after incarceration [3, 7]. The burden is much higher in prison populations that are associated with resource-limited countries [3, 8]. A prevalence of greater than 4% has been documented in Ethiopian prisons [9], which is more than four times higher than the prevalence in the general population – one of the highest HIV prevalence in prison populations in sub-Saharan Africa (SSA) relative to the general population [7]. Poor ART adherence is common in inmates living with HIV (ILWH) both in high- and low-income countries [10–13].

Structural, psychosocial and behavioural factors have contributed to poor ART adherence in ILWH. Protracted institutional processes involved in accessing care [11, 14–16], unplanned transfers between correctional facilities [15, 17] and poor care provider support [15, 16, 18, 19] constituted the structural circumstances. A lack of social support [18, 20–24], stigma related to loss of privacy [13–16] and having a psychiatric disorder [16, 21, 22, 24, 25] are factors related to the psychosocial component. The level of ART adherence is often low in ILWH who have a history of injecting drug use [16, 23, 24] and in those who adopt negative perceptions of the safety and efficacy of ART [22, 23, 25].

There has been only limited information available regarding contextual factors influencing ART adherence amongst ILWH in resource-limited countries. An absence of standard HIV care in the prison systems and associated barriers including: a lack of transport to ART sites external to prison [13, 26, 27], as well as an insufficient supply of food [13, 19, 27] predominate the available evidence. However, it is unclear how ILWH access HIV care and use medication at different trajectories of the incarceration process and what circumstances influence this from the perspectives of ILWH and the relevant stakeholders. Obtaining a comprehensive understanding of the situation would help establish a ground for development of contextually-responsive intervention strategies beyond contributing to closing perpetuating policy gaps in the equitable distribution of resources for the criminal justice system [28]. We undertook a qualitative exploration of barriers to and facilitators of access to HIV care and medication use amongst ILWH in South Ethiopia – a resource-limited setting with a steady HIV prevalence despite a steep decline at national level, attributed to its majorly rural communities [29] from where prisoners often originate [30].

## Methods

### Study setting

A detailed description of the study setting is provided in our earlier paper [31]. In summary, we conducted in-depth interviews in four selected prisons - Hossana, Wolayta, Wolkite and Worabe prisons in South Ethiopia and in the respective external ART clinics that were providing HIV care for ILWH in the prisons. All the prisons serve both male and female prisoners in separate units. The four prisons had a daily average number of about 3,635 inmates, with an average daily entry of 15 persons in each prison [32]. There has not been any specification for the correctional facilities regarding the level of security or holding capacity.

### Participants

Eight male and three female ILWH were interviewed, as were eleven service providers (seven male and four female service providers). ILWH participants who were 18 years or older were selected purposively based on length of incarceration and experience of HIV care use for at least six months. These ILWH were assumed to have sufficient institutional experiences with which to generate dense and focused information on structural, social and personal barriers to accessing care in the prison context. In addition, ILWH were also required to have been fluent in Amharic language in order to maintain verbal fluency and clarity of ideas to the researcher who conducted the interview [33, 34].

Among the service providers interviewed were: two prison health staff, three ART service providers, two prison officers, two prison administrators and two health agents. Service provider participants were selected based on their role in HIV care provision for prisoners. The prison health staff interviewees were engaged in providing routine medical care for prisoners, whereas ART service providers were health professionals who were providing ART services for both incarcerated and non-incarcerated PLWH at the selected public health care facilities. The prison officers were involved in accompanying ILWH to external ART clinics to access care. The prison officials interviewed were members of the administrative bodies of correctional facilities, whereas the health agents were representatives of the respective Zonal Health Departments who technically support the prison healthcare system. All staff participants had more than six months of experience in their respective positions.

The sample size for both groups was determined based on theoretical saturation. This was detected when no new concepts emerged while participants were recruited sequentially to ensure representativeness and diversity with regard to a range of experience (in HIV care use at different trajectories of the incarceration process for ILWH participants and facilitation and care provision for service providers), prison settings and role in the provision of care [33]. Participants were offered a compensatory payment for their time commitment taking into account the amount of time they devoted and their daily earning background.

### Data collection

The principal researcher (TGF) conducted the interviews using an interview guide constructed with open-ended contextual questions as described in the earlier paper [31]. The interview guide for prisoners focused on structural, social and behavioural contexts promoting and hindering HIV care use in the prison system. The structural aspect addressed issues related to access to standard HIV care and institutional contexts influencing this. While the social component addressed both encouraging and discouraging social circumstances from inside and outside prison, personal contexts focused on inmate’s understanding and perception of ART use in the prison environment. The service providers were asked for their experiential account of the existing HIV care provision and support strategies. This added diverse points of view to prisoners’ perspectives on HIV care use in the prison environment.

The interview guide was piloted with people from the targeted population (two for ILWH participants and one for each category of service provider participants) at institutions other than the study sites. This allowed identification of elements which supported the objectives of the study, inclusion of relevant concepts which had not been considered previously and modification of those which were found to be incomprehensible to the participants.

For both groups of participants, the interviews were undertaken in Amharic language, a widely spoken language across Ethiopia and in the study area. Prisoners were interviewed (45–60 min) in a private secured place in a prison clinic. Due to security concerns in prison settings, prison health staff guided the principal researcher to contact the prisoners in order to obtain consent for voluntary participation when they made their regular clinic visits. To facilitate this, clinic appointments of all eligible prisoners were initially retrieved from medical registers. However, the health staff played no role during consent and interviewing processes.

Service provider participants were identified by contacting the prison and health administrations. The study eligibility criteria were presented to the respective administrations in order for them to help the principal researcher find potential participants. All service providers were approached during their office hours and interviewed (40–50 min) in their respective offices in private, after they gave informed consent for participation. The interviews were audio recorded and field notes were made on tacit knowledge [35, 36]. The principal researcher initially transcribed the audio recorded interview data in Amharic language and then translated into English for analysis.

### Analysis

We employed a descriptive phenomenological approach so as to abstract meanings attributed to the lived experiences of the participants regarding access to and use of HIV care in the prison context as presented to our consciousness [37, 38]. We first read the whole raw data (transcripts and field notes) to understand the basic sense of the experiences described. The descriptions were then broken into parts based on the meaning they had (‘meaning units’) with respect to access to and use of HIV care. This was achieved by determining a transition in meaning while reading and re-reading the descriptions.

For example, a prisoner at some point of his description stated, *“You know what, when you go to the health centre regularly, people become suspicious* [of being HIV infected]. He then went on to say, *“I haven’t disclosed to anyone. I was imprisoned in this prison before, and there was a guy from another town, because he has HIV in his blood, no one wished to have a meal with him.”* While the first description holds meaning regarding challenges in keeping one’s privacy (regarding HIV status) in the prison context, the second one relates to the influence of enacted stigma on disclosure, which also represents the psychological meaning of the experience.

The principal researcher (TGF) initially coded and recoded the meaning units to check if there was any intrapersonal inconsistency in the coding process. The researcher used NVivo12 qualitative data analysis software [39] to code and juxtapose the meaning units in a chronological order of events and conceptual relationships. Final meaning units were decided after triangulating different interpretations and reaching a consensus between the researchers (TGF, GT and ERM) through subsequent discussions and review of the descriptions. Participants were provided with the summary of the results and asked to verify the accuracy of the results when data interpretation was completed [40] and all participants verified the accuracy and agreed with the results. Finally, the general structure of the experiences was described by pooling and comparing supporting and opposing concepts (psychological meaning units) within and between transcripts in terms of recurrence, patterns and relationships [35, 38, 41, 42].

Reflexivity was considered important for the analysis regarding the influence that the principal researcher may have had during his interactions with participants. The researcher belonged to the same ethnic background and shared many of the same cultural practices from which most of the prisoner participants originated, which might have partly given him an insider role to access the culture and ask participants more meaningful questions [40, 43]. The potential difference in socioeconomic and educational status between the researcher and prisoner participants might have impacted the trustworthiness of data. This could be in association with the situation that most prisoners in South Ethiopia come from impoverished rural settings with little educational access [30] which could potentially limit their ability to have a close vicinity to the researcher and candidly express their feelings. Nevertheless, the researcher’s previous research experiences in the same settings [30] offered an opportunity to understand the research context [40, 43]. The researcher constantly maintained a journal of the research process encompassing experiences, emotions and change in attitudes towards participants and how this could impact data [44]. This helped bracket all knowledge coming from natural attitude (e.g., the researcher’s pre-existing attitude of the prison environment in relation to health) and concentrate on the givens presented to his consciousness [38].

## Results

### Participant characteristics

The characteristics of ILWH participants are presented in Table 1. ILWH participants had a median age of 35 years (Interquartile range (IQR): 30–45 years). Most (64%) of the ILWH reported elementary school (1-8th grade) as their highest educational attainment. Eight inmates had been incarcerated for more than one year, six were diagnosed with HIV during incarceration, and seven were initiated on ART in prison. Six inmates reported five or more years’ experience of living with HIV and five had used ART for five or more years.

**Table 1:** Demographic, incarceration and HIV care-related characteristics of prisoner participants

Participant	Site	Sex	Age range (Code)	Education	Length of time in prison (years)	Time since HIV diagnosis (years)	Time on ART (years)
P#1	A	M	2	Primary	1	7	6
P#2	B	M	2	Secondary	5	4	4
P#3	B	M	2	Secondary	9	9	8
P#4	B	M	1	Primary	3	7	7
P#5	C	M	3	Primary	4	5	4
P#6	C	M	2	Secondary	5	12	12
P#7	C	M	2	Primary	0.7	0.7	0.7
P#8	D	M	2	Primary	0.5	0.5	0.5
P#9	B	F	4	Primary	4.5	4	4
P#10	A	F	3	Primary	9.5	11	10
P#11	B	F	2	NE	3.5	1.5	1

ART: Antiretroviral therapy; F: Female; M: Male; P: Prisoner

Site: Indicates a prison; Primary education: 1-8th grade; Secondary education: 9-12th grade; NE: No formal education

Codes for age range in years; “1”= 20–30; “2” = 31–40; “3” = 41–50; “4” = >50

Details of the characteristics of service provider participants are presented in Table 2. All prison health staff and ART service providers had a tertiary qualification in health related disciplines and more than six months’ experience in their respective positions. Prison officer participants had two or more years’ experience of facilitating ILWH’s accessing of care from external ART clinics. Prison and health administrator participants had been managing and providing technical and material support for the prison healthcare system for four or more years.

**Table 2:** Demographic details, military rank and HIV care-related role of service provider participants

Participant	Site	Sex	Age range (Code)	Education/ Military rank	Experience on HIV care (years)	Role in prison HIV care
PN#1	B	F	2	BSc(Nurse)	10	Testing and linkage to care
PN#2	C	F	1	BSc(Nurse)	9	Testing and linkage to care
AP#1	A	F	2	BSc(Health Officer)	4	ART initiation /Adherence support
AP#2	B	M	3	BSc(Health Education)	5	ART initiation /Adherence support
AP#3	C	F	1	BSc(Health Officer)	0.6	ART initiation /Adherence support
PO#1	B	M	2	College graduate (Sergeant)	2	Guarding during clinic visit
PO#2	C	M	2	Secondary (Deputy Inspector)	10	Guarding during clinic visit
PA#1	B	M	2	BA(Economics/Assistant Inspector)	14	Prison Head
PA#2	C	M	3	College graduate (Commander)	11	Prison Head
HA#1	A	M	3	BSc (Health Officer)	4	Zonal HIV Coordinator
HA#2	C	M	4	BSc (Nurse)	5	Zonal HIV Coordinator

ART: Antiretroviral therapy; AP: ART service provider: BSc: Bachelor of sciences; F: Female; M: Male; HA: Health agent; PA: Prison administrator; PN: Prison nurse; PO: Prison officer

Site: Indicates a prison or a health department or a health care facility offering ART services; Secondary education: 9-12th grade

Codes for age range in years; “1”= 20–30; “2” = 31–40; “3” = 41–50; “4” = >50

The following section presents various structural and social contexts that emerged as barriers to and facilitators of HIV care use amongst ILWH in South Ethiopia. Under each identified concepts are selected quotes that exemplify the reflections of most participants. Pseudonyms are used instead of real names of individuals mentioned in the interviews and letters to represent the prisons, health care facilities as well as health departments in order to prevent potential identification of persons providing the information.

### Barriers to HIV care use

#### Structural context

##### Limited access to care

The prison system in South Ethiopia was marked by a lack of standard HIV care which imposed additional suffering on ILWH and represented a ‘double burden’, the imprisonment itself and inappropriately treated HIV infection. The perception of many of the inmates’ was that both HIV infection and incarceration occurred incidentally but were highly likely to produce psychological as well as physical trauma. One prisoner who used ART in prison for four years explained how difficult it was for ILWH to cope with both conditions in a context where there was sub-optimal HIV care:

“Most [HIV infected] people suffer here because of lack of care; they are embarrassed by imprisonment on one hand and by the disease on the other hand.” (Male prisoner, age: ‘2’; Prison ‘B’).

Although ILWH who had been using ART before incarceration showed an intention to continue using the therapy after being incarcerated, absence of HIV care in the prison system caused substantial delays in treatment continuation.

“I thought I had enough in my bag [during arrest] but it was empty. Then I let them [prison health staff] know about the issue but there is no HIV treatment service here.” (Male prisoner, age: ‘2’; Prison ‘C’).

In all prison settings, ILWH were accessing ART services from external health care facilities, which presented a series of institutional and inter-institutional barriers to care. At some settings, access to care was adversely influenced by the long distance between a prison and an ART site, as there was no facilitation of transport by the prison system.

“It [referring to an external ART site] is too far to go on foot. Sometimes it feels demotivating because of the exhausting journey.” (Female prisoner, age: ‘3’; Prison ‘A’).

A shortage of prison officers caused delays in ILWH’s health care facility visits as they were forced to go *en masse* even if their appointment fell on different dates:

“There are times that they [prison health staff] jump our appointment. Sometimes there could be even a shortage of guarding police.” (Male prisoner, age: ‘2’; Prison ‘B’).

Among inter-institutional factors, poor organisational relationships between prisons, health care facilities as well as courts presented a substantial barrier to care. A lack of collaboration between health care facilities and the correctional system resulted in interruptions of ART during prison entry and transfer of ILWH between correctional facilities. A prisoner who had been using ART before incarceration discussed his experience of the challenges of pursuing ART during prison entry:

“---Then the hospital on its turn said, ‘We don’t treat him unless we receive a referral!’ I just remained without medication in the middle.” (Male prisoner, age: ‘2’; Prison ‘A’).

ART service providers described the difficulties in tracing back records of newly arriving ILWH who had been using ART elsewhere. As prisoners were often transferred abruptly, ILWH often had limited opportunity to arrange consultations with ART service providers and complete their pre-transfer medication requirements:

“---Because he [newly arriving ILWH] often comes suddenly; For example, they [prison staff] are not going to ask him when he will be leaving so that he can collect his medical information from the Hospital. They just pick him up and transport to somewhere.” (Female ART service provider, age: ‘2’; Health facility ‘A’).

Health care facility and court appointment times sometimes overlapped, leaving ILWH oscillating between the two, with no power to influence a change for either appointment:

“---Then I got troubled when the hospital appointment overlaps with the court appointment. When I ask the judge to change the appointment, he says, ‘Are you the judge? Then when I inform them [prison health staff], they say, ‘Why didn’t you go there [to the hospital] yesterday? Wasn’t your appointment yesterday? I say, ‘I went to the court!’” (Male prisoner, age: ‘2’; Prison ‘A’).

This situation led ILWH to perform medically discouraged acts such as using unprescribed medications borrowing from fellow inmates. The aforementioned inmate went on to describe the negative impact of the circumstance on his medication use:

“---We also used to borrow [meds] from each other here; with ‘Usman’ and ‘Feleke Girma’. Sometimes the health facility appointment overlaps with court appointment.” (Male prisoner, age: ‘2’; Prison ‘A’).

The health administrators tended to accept that ILWH accessed ART services from external health care facilities as normal practice, on the basis of perceived difficulty of introducing ART services within the prison system:

“---So, going to the hospital is the only option they [ILWH] have and it doesn’t matter if they go there every month; no other option!” (Male health agent, age: ‘3’; Zonal Health Department ‘A’).

##### Insufficient health staff support

Prison health staff appeared to feel little responsibility towards ILWH given HIV care operated entirely external to the prison healthcare system. Both ILWH and service provider participants discussed the deficiency of care provided to ILWH via the prison healthcare system. As one prisoner stated, prison health staff were not considered central to HIV care in the prison system:

“There is nothing we obtain from them [prison health staff]; they just send us to the hospital based on our appointment. Our contact is with the hospital staff not with them.” (Male prisoner, age: ‘2’; Prison ‘B’).

Prison officials discussed the challenges that they faced in making referrals because of ILWH’s lack of confidence in the prison healthcare system. They suggested an imbalance between the community and prison healthcare systems in terms of resource allocations:

“---We are now having trouble making a referral. So a work position which encompasses doctors should be designed. There is one at a hospital; there is one at a health centre; why is it limited for correctional facilities?” (Male prison administrator, age: ‘2’; Prison ‘B’).

In acknowledging complaints made by the prison officials about inadequate health staff training, health administrators tended to blame higher health agencies, such as the Regional Health Bureau, for their inequitable delivery of professional training between the community and prison healthcare systems:

“They [prison administrators] often complain about their health staff being neglected [in terms of professional training]; ---even the Regional Health Bureau gives more focus to public health care facilities. We have recently implored them a lot to train the health professionals there [at prison]. ” (Male health agent, age: ‘4’; Zonal Health Department ‘B’).

Because of limited communications from ART service providers at public health care facilities, prison health staff lacked knowledge about how effectively ILWH were using their medication:

“They [ART service providers] never give us feedback. I don’t actually know whether the prisoners’ CD4 count is declining or viral load rising.” (Female prison health staff, age: ‘1’; Prison ‘C’).

ART service providers also described an almost complete lack of communication between themselves and prison health staff; one even indicating that their knowledge of prison health staff was solely derived from their participation in the current study:

“--- It was your study that made me know that girl [a prison nurse], her phone and even about the correctional institution itself; I have never thought of it.” (Female ART service provider, age: ‘2’; Health facility ‘A’).

The ART service providers’ limited understanding of the context of HIV care for ILWH may have contributed to the low HIV care uptake among ILWH at some external health care facilities. ILWH commonly discussed the need for self-motivation in managing their treatment as they were only rarely receiving adherence support from ART service providers:

“---I was provided with the counselling service the first day. I have been striving by myself since then. I’m using it just to see tomorrow. But [Clapping his hands] I haven’t received any advice since then.” (Male prisoner, age: ‘2’; Prison ‘C’).

##### Uncooperative security systems

Interruptions to medication use occurred at different stages of the incarceration process due to uncooperative security systems. One prisoner who had started ART before imprisonment discussed being prevented from accessing his medication during arrest and the delays that subsequently occurred in his medication continuation because of the absence of ART services in the prison system:

“I told this to her [an ART service provider]; the police denied having my medication with me during arrest. I was arrested suddenly by the police on my way to home. I implored them, ‘I’m a patient, let me just have my meds with me!’ ‘You’ll take medicine from the hospital there!’ they replied. ‘I did not take the meds for three days.” (Male prisoner, age: ‘2’; Prison ‘A’).

ILWH’s medication use after prison entry was also constrained by protracted security processes. Incoming ILWH were obliged to remain without medication until the police investigations were completed about any medications they may have had with them on arrest:

“I was troubled the first day [during entry]. It was because the security had taken away my drugs for a check by the health staff, and this took a while.” (Male prisoner, age: ‘2’; Prison ‘C’).

In addition to the sense of a diminished responsibility towards ILWH exhibited by prison staff, prison security presented an additional barrier to accessing supplementary medications available in the prison system:

“He is a treatment facilitator. He is the one who lets us see her [a prison nurse]. It is really hard even to come here! Oh, I can’t come here if I have some stomach-ache, and she knows this. I gently explain to him this because he should not be offended.” (Male prisoner, age: ‘2’; Prison ‘A’).

Prison security related factors also impacted the quality of care delivered by ART service providers. Prison officers’ often allowed insufficient time for ILWH-ART service provider discussion and laboratory investigations, which influenced ILWH’s care use:

“---Even after they have arrived at here [an ART site], they cannot receive appropriate support and laboratory results like other clients. The guarding police are unwilling to go out for us to talk privately with the clients.”(Female ART service provider, age: ‘2’; Health facility ‘A’).

One ILWH witnessed a prison officer’s intrusion in a client-health care provider discussion and the argument that resulting in ILWH being taken away from the health care facility before their consultations were completed:

“They [prison officers] don’t allow us to stay there. She [an ART service provider] insulted them sometime and told them, ‘Don’t do this, you may also be arrested tomorrow!’ If in case treatment delays, they just rush us off saying, ‘Let’s go!” (Male prisoner, age: ‘2’; Prison ‘A’).

##### Loss of patient privacy

Both ILWH and service provider participants discussed challenges in relation to keeping patient privacy and confidentiality within the context of the prison system. Congregated living conditions and regular external ART visits (often guarded *en masse*) contributed greatly to loss of privacy. A prisoner who had been taking ART in prison for six years explained circumstances when patient privacy was affected for ILWH in relation to their HIV status:

“---You know what happens in this [prison] compound, he [HIV infected prisoner] may attempt to hide for some time, but people will make it overt soon. Since we live in the same compound, every prisoner is aware of who has the virus and who doesn’t.” (Male prisoner, age: ‘2’; Prison ‘C’).

One prisoner who had not previously disclosed his HIV status described his experience of having his medication divulged during external ART visits, and his struggle to keep his diagnosis confidential. Prison staff and fellow inmates became suspicious of his being HIV infected because of his regular external ART visits and security checks at the gate:

“You know what, when you go to the health centre regularly, people become suspicious [of being HIV infected]. And in your check-in, prison security fumble into your pocket and may bring the meds out.” (Male prisoner, age: ‘2’; Prison ‘D’).

Patient privacy was also impacted by casual communications between prison health staff and ILWH. Prison health staff were unable to maintain patient privacy, which was challenged by the highly exposed prison environment. An undisclosed HIV-infected prisoner shared his lived experience of loss of privacy during contact with a prison nurse:

“---Rather patient privacy is severely divulged here in the prison. One day he [a prison nurse] called me and asked ‘Where are those who got diagnosed with you?’ There were a lot of prisoners around me when he was saying this. I was so embarrassed. It should be me and him who should know about this, isn’t it?” (Male prisoner, age: ‘2’; Prison ‘D’).

As correction staff responsible for accompanying ILWH to ART visits were frequently changed, inmates’ HIV status was constantly disclosed to different people at different times without consent. One ART service provider argued for specified prison officers to escort ILWH to alleviate the problem of privacy loss at least to some extent, recalling similar situations from his previous experience to support the claim:

“It is not just one person who knows them. What I heard in ART training was that clients complain about frequent changing of cleaners; Why not only one person? You see the case of the guarding police? They are different prison officers who bring them at different times.” (Male ART service provider, age: ‘3’; Health facility ‘B’).

##### Insufficient food supply

Almost all ILWH participants reported insufficiency and poor quality of food in the prison system. Most of them perceived that the poor quality of food caused more suffering to those who were using ART. Anticipating its possible negative effects on the health benefits of the medication, an inmate favoured taking the medication before a meal, which was against instructions:

“---The drugs and the food we eat are totally irrelevant, particularly the bread…it looks like something made of mud! I take my medication on empty stomach fearing that it may provoke nausea. Although unpleasant, it is better to eat it after taking the drugs unless it may affect the effectiveness of the drugs.” (Male prisoner, age: ‘2’; Prison ‘C’).

ILWH reported being challenged to maintain their medication use, often ascribing their vulnerability to drug side-effects to their impaired physical states produced by insufficiency of food. They felt uncertain about being capable of pursuing the use of medication in the face of combined adverse effects of poor quality food and the medication itself:

“Sometimes I skip the meds when I feel empty stomach after having eaten this dry loaf of bread… It makes me like fatigued.” (Male prisoner, age: ‘2’; Prison ‘A’).

ART service providers experienced a challenge while verbally persuading their prisoner clients to adhere to the medication instructions, as ILWH held poor self-efficacy to pursue the course of action because of the food:

“There are individuals [ILWH] who complain about the food provided by the prison and say, ‘We’re almost burned, the meds are burning us! We’re taking this medicine with that food!” (Male ART service provider, age: ‘3’; Health facility ‘B’).

Prison officials failed to respond to this, although they often did recognise ILWH had greater dietary requirements than ordinary inmates because of their medication use. They often blamed funding agencies for their limited budget allocation for necessary additional food rations for ILWH:

“--- We see them [ILWH] as prisoners. It is 0.7USD [United States Dollar] a day, 20USD a month. There is no exception to him; he is fed in the same manner as other inmates are. Of course, it’s not enough. First, HIV patients should get balanced diet…It is problematic that there is no any additional support other than the normal budget allotted for other inmates.” (Male prison administrator, age: ‘3’; Prison ‘C’).

At the community level, while there had been a substantial number of supporting agencies outside prison working on nutritional issues of PLWH, the prison system seemed to be in isolation from such programs as it lacked due attention from these agencies:

“Non-governmental organisations such as What-sup, Nastad and others too, they are about three, and they carry out supporting works in the community. They give them [PLWH] money and grain. But if the [prison] clinic had some social linkage, they would have supported them as well.” (Male ART service provider, age: ‘3’; Health facility ‘B’).

#### Social context

##### Social stigma and HIV status disclosure

Some ILWH who had been using ART before incarceration felt unable to disclose their HIV status and/or their previous use of ART to prison staff and were therefore more likely to discontinue their medication. A prison officer who often took ILWH to external ART sites came across such a situation:

“…There are people who are aware of their HIV status prior to prison entry and got into without disclosing their status. However, it will eventually be revealed when his health condition gets worse.” (Male prison officer, age: ‘2’; Prison ‘C’).

ILWH were discouraged from disclosing their HIV status because they had observed that it resulted in adverse consequences such as enacted stigma to those who had performed the course of action. One prisoner said that he preferred to keep his HIV status secret to avoid potential maltreatment by his fellow inmates:

“Yes, I haven’t disclosed to anyone. I was imprisoned in this prison before, and there was a guy from another town, because he has HIV in his blood, no one wished to have a meal with him, they let him eat alone. They prohibited him from using water and tea utensils together.” (Male prisoner, age: ‘2’; Prison ‘D’).

Perceived stigma dissuaded ILWH from disclosure due to its possible adverse consequences such as emotional suffering, which might prevent them from participating in prison social life:

“Nothing but a psychological trauma [if disclosed]; You know, these are uncontrolled people. When they call you, ‘You ill!’ you may feel embarrassed, tortured and get annoyed all day.” (Male prisoner, age: ‘2’; Prison ‘D’).

The effect of stigma on ILWH appeared to be even more intense when it was enacted by prison officers, often leading to despondency:

“You know they [prison officers] consider me as dead: they perceive me as if I carry death like a laptop. I’d love to see if they are provided with a sort of training and I wouldn’t be tortured mentally.” (Male prisoner, age: ‘2’; Prison ‘A’).

Nonetheless, prison health staff and health agents reported that incoming ILWH should have announced their HIV status to prison staff to ensure continuation of their medication. They appeared to possess diffused responsibility for offering ILWH appropriate professional support. A prison nurse explained that she wasn’t in a position to help incoming ILWH if they were unable to inform her about their HIV status:

“---However, if there is someone who is arrested and doesn’t speak to us, we can’t help. We haven’t yet examined them while entering.” (Female prison health staff, age: ‘2’; Prison ‘B’).

Incoming ILWH who felt able to disclose their HIV status and/or their previous use of ART to prison staff were less likely to experience medication interruptions that often occurred during prison entry. One female inmate who had been on ART before imprisonment reported her lived experience regarding this:

“After I told the security at the police station that I had a prescription, my family brought me my medication card, and then I went to the hospital with the police to continue my medication.” (Female prisoner, age: ‘3’; Prison ‘A’).

Some inmates believed that their capability to disclose their HIV status dispelled possible embarrassment and enabled them to confidently use their medication, as well as to interact with members of the prison communities. It helped them obtain peer support essential to manage their medication use within the context of custodial settings:

“The time a guy died of HIV; while I was playing with one of my friends, two guys came to us, and I heard one of them saying to my friend like, ‘Take care not to be pricked by that guy’s nails!’ Even I myself got embarrassed at that moment [Laughs]! And I just went away laughingly; I never get embarrassed because everyone knows about it. I just talk to everyone; I don’t hide it! Why would I? I even ask them to tell me the time to take my medication as my watch is not working now.” (Male prisoner, age: ‘3’; Prison ‘D’).

ILWH who were able to accept their HIV status were capable of coping with privacy affecting prison environments and demonstrated a high level of confidence to pursue normal life while using the medication. Their self-confidence enabled them to also cope with negative responses from others in regard to their HIV status:

“I can’t do anything even if it [stigmatisation by fellow inmates] exists, I just ignore it. They can say whatever they want to say, I don’t care about that.” (Female prisoner, age: ‘2’; Prison ‘B’).

### Facilitators of HIV care use

#### Structural context

##### Prison as a facilitator of HIV care use

Imprisonment encouraged some PLWH to refrain from behaviour which adversely affected their ability to appropriately use care. One prisoner reported:

“I started taking the meds properly after I became imprisoned. When I was out there, I used to smoke forty cigarettes per day; I also used to chew “khat”. I was so desperate! Since I came here, I just started to think about and wondering what was going on; refrained myself from being with addicted friends, my reduced CD4 [count] shown some gains as I have got checked for it last time.” (Male prisoner, age: ‘2’; Prison ‘A’).

ART service providers observed some behavioural changes amongst their clients during incarceration with significant improvements in treatment outcomes. They tended to relate this to less accessibility of undesirable social networks that existed outside prison:

“Sometimes I see incarceration as an opportunity. There were clients who had behavioural problems, and addicted to substances like cigarette and had an adherence problem. However, after they had got into there [prison], they don’t think of other things, they only think of their medicine; there are people whose adherence has improved. Yes, their viral load and CD4 count are better; even better than those in the outside community.” (Female ART provider, age: ‘2’; Health facility ‘A’).

Imprisonment was found to offer conducive social environments for ILWH to learn about the adverse consequences of medication interruption from peers who had experienced these consequences:

“There were persons whose feet got paralysed and had some inflammation on their body [due to medication interruption]. Then I say, ‘What’s the matter if I take this little thing [the medication]! Isn’t this easier than what I have every time?’” (Male prisoner, age: ‘2’; Prison ‘B’).

#### Social context

##### Social support

Prisoners described the importance of social networks for enhancing care use in prison. In their view, social networks serve as a means for ILWH to offer each other information, material and emotional as well as affectionate support. Through social networks, senior ILWH would encourage incoming ILWH to disclose their HIV status in order to access care and support. An inmate who had been using ART in prison for four years reported the benefits that ILWH used to gain from such a network:

“---We used to help each other when there was anyone who is seriously sick; we used to give him money from our common account. If it was in the past, they [incoming ILWH] would report to the club, and then we would let the concerned body know that there are new arrivals. We would receive them kindly.” (Male prisoner, age: ‘3’; Prison ‘C’).

The social networks also functioned as a means to earn income for accessing supplementary food and transport to external ART sites. It fostered cooperation amongst ILWH to run various business activities otherwise might be difficult to carry out individually in a prison environment:

“We had our own barbershops, table tennis and other stuff; we made monthly meetings. They would have allowed us to run our club so that we could have covered our transport costs.” (Male prisoner, age: ‘3’; Prison ‘C’).

ILWH also believed that social networks have the capacity to dispel dejections, and avoid alienations by HIV non-infected prisoners:

*“At the time we had the club, we used to get together and talk to each other about our daily problems. When we did that, we got a sense of relief. But now, everyone particularly the healthy ones point their finger at you.” (Male prisoner, age: ‘3’; Prison ‘C’).*

Notwithstanding the value of the social networks for ILWH, some prisoner participants reported a denial of the resources that used to be offered in their prison, which were essential for the existence of the associations. Participants related this to unresponsive prison officials who undermined the seriousness of HIV infection:

“We asked them [prison administrators] to allow us to run the club by ourselves. They denied it although we have the capacity to work. Someone in the office once ironically responded, ‘You call this [HIV] a disease? This is a kind of an ordinary illness!’” (Male prisoner, age: ‘3’; Prison ‘C’).

ILWH also tended to blame health agencies for the decline in care and support at the prison system including social networks:

“The anti-HIV club should have been closely monitored with governmental and non-governmental organisations taking part. I don’t think the club is recognised by the government; it rather seems that people voluntarily support us. I don’t think the government recognises that there are people living with HIV in prison; whether we are alive or dead.” (Male prisoner, age: ‘2’; Prison ‘C’).

There was an inequitable distribution of resources important for the establishment of such social networks between incarcerated and non-incarcerated PLWH; the former being devoid of health agencies’ due attention. One ART service provider described the alienation of PLWH at prison settings from various social activities that were undertaken in the community, which served as an important point for dissemination of information for affected people:

“They [prisoners] are very isolated in this perspective. Because there are training sessions that I often go and offer; HIV positive people will be called and get organised, and provided with [life skills] training. So, they have a very slight chance of getting training compared to people in the outside community. I mean there is no one who thinks there are [HIV] patients at prison [Laughs]!” (Female ART service provider, age: ‘2’; Health facility ‘A’).

Health agents acknowledged that the benefits of social networks had been clearly demonstrated in the community settings for PLWH. They were operating as a means to gain government agencies’ attention and build self-confidence among PLWH to share each other their lived experiences, and run various income generating activities. However, the health agents recognised the scarcity of such social networks at the prison system, ascribing this to the instability of prison populations and restrictions inherent to prison settings:

*“There are associations related to HIV in the Town. They have an association so that they have nothing to fear; they stand in front and teach; they trade like a normal person. The situation of prisons is quite different; they are a little strict* [Laughs].*” (Male health agent, age: ‘3’; Zonal Health Department ‘A’).*

## Discussion

Using lived experiences of prisoners and experiential accounts of service providers, this study sheds light on circumstances that influence optimal use of HIV care in people involved in the criminal justice system in South Ethiopia. It created understanding of how HIV-infected prisoners (one of the HIV key population groups worldwide) access care in resource limited contexts from different perspectives in order to establish a ground for developing context-responsive intervention strategies. The views of both groups of participants concurred that as well as social and individual level factors, prison system structural factors appeared to play a crucial role in determining ILWH’s ability to appropriately use care. Overall, there was a lack of socially agreed principles that justify appropriate distribution of healthcare resources between the prison and community healthcare systems, leading to sub-standard HIV care in the prison system. Prisoner participants perceived the situation as ‘a double burden’ of being imprisoned and having inadequate HIV care. The interplay between these ‘burdens’ exacerbated the adversities faced by ILWH.

Implementation of standard HIV care programs in prison is recommended by international guidelines [45–47] and has proven to be feasible and effective both in high- and low-income countries [48–50]. This study strengthens previous evidence that most countries in sub-Saharan Africa (SSA) lack comprehensive policies supporting this recommendation [51, 52], which may lead to sub-optimal treatment outcomes and facilitation of community transmission [1–3].

Most institutional and inter-institutional barriers presented by external ART services in the current study are commensurate with findings of other investigations in low-income countries [26, 27]. A lack of transport facilities combined with shortage of escorting prison officers led to group clinic presentations, which in turn caused frequent missing of clinic appointments, and use of unprescribed medications. Regular visits to external ART sites in a group and a frequent shifting of guarding prison officers severely affected patient privacy (for undisclosed ILWH) which is already challenged by congregated living conditions in a prison environment [13, 15].

Participants reported medication interruptions at various stages of the incarceration process, which related to the uncooperative security system. Denial of medication possession during arrest coupled with protracted security processes during prison entry forced incoming ILWH to interrupt their medication. After prison entry, prison officers’ obstruction of ILWH-ART service provider consultations influenced ILWH’s care utilisation and caused emotional trauma and demotivation of reporting medical concerns. These findings are consistent with what Shalihu, Pretorius (13) reported in a Namibian prison, where prison officers’ discriminatory threatening of ILWH caused “frustration, humiliation and discouragement” in relation to ART use (page 971). Other studies have also described frequent medication interruptions amongst ILWH due to uncooperative prison security even where on-site ART services are available [11, 15, 16]. This underscores the imperative of creating awareness amongst security staff regarding the necessity of HIV medication for ILWH during every step of the incarceration process, as well as the importance of health care provider counselling and support for maintaining optimum care use [53].

There was insufficient health staff support for ILWH both from the prison healthcare system and external health care facilities. The inadequacy of prison health care staff support was mostly derived from a lack of HIV-related training and possession of dissociated responsibility for ILWH (given HIV care was provided externally), as well as poor communication with ART service providers. This drove ILWH to have reduced trust in prison healthcare system and instead rely on the external health care services, despite these services being largely inaccessible. Research shows that adoption of the belief that health care providers are uncaring and unsympathetic negatively affects care use [15].

HIV care provided to prisoners by external ART clinics was also found to be sub-optimal. This was demonstrated by a lack of continuous counselling and support during and after initiation of ART. Optimal health care provider support is crucial for maintaining ART adherence in ILWH [15, 16, 18], however, the inadequacy of trained health care staff remains a challenge in many prison systems [11, 14, 15]. Thus, provision of HIV-related training for prison health care staff is highly recommended in addition to strengthening communication between prison and community healthcare systems [54].

According to the service providers, ILWH were required to disclose their HIV status and/or previous use of ART to prison staff if they were to continue their medication. However, this study and others [11] found that ILWH often lack the confidence to disclose their HIV status to prison staff, often disclosing only when their health worsened due to the progression of infection. Vicarious and direct experiences of social stigma by fellow inmates and prison officers played a role in hindering ILWH’s motivation to disclose their status, and so lessened their commitment to use care. The impact of debasement was more intense when enacted by prison security often leading to despondency, the main predictor of sub-optimal care use in incarcerated people [16, 21, 22, 24, 25]. This supports the well documented negative influence of marginalisation and discriminatory treatment on the basis of HIV status on ILWH’s care use [13–16].

Despite the absence of organisational structures supporting disclosure and ensuring patient privacy in the prison system, ILWH who felt able to disclose their HIV status were able to reduce potential medication interruptions, consistent with previous findings [11], and to create sources of social support. Disclosure also served a means to gain self-confidence important to cope with social stigma, and internal satisfaction and motivation to support oneself and others in a similar situation, as Sprague, Scanlon [55] puts it “generating a type of solidarity” (page 1437). This implies a need for interventions that enhance consented disclosure amongst ILWH, while preserving patient privacy and confidentiality; increasing access to HIV counsellors and reducing social stigma through improving general understanding of HIV amongst prison staff and prisoners may facilitate disclosure [56].

In prisoners’ accounts, imprisonment appeared to be more burdensome to ILWH than their non-HIV infected counterparts in the face of food supply insufficiency. This is because the former group use a therapy (ART) that requires a higher quality diet [57]. The insufficient quantity and quality of food aggravated medication adverse-effects which often predicts poor adherence in prisoners [16, 17, 19, 20, 22]. The influence of food insufficiency on ART adherence in this study is consistent with findings of studies in SSA [13, 27] and other low- and middle-income countries elsewhere [19, 58], which reported frequent missing of doses and treatment interruptions amongst ILWH due to hunger. Efforts should be made to enhance food support programs in prison settings and give special focus to ILWH in the nutritional programs designed to support PLWH at public health care facilities [59].

The social networks of ILWH were found to operate as an essential source of social support including instrumental, emotional and information support, as well as creating a sense of comradeship. The social establishment also acted as a means to gain vicarious experiences related to medication use, avoid social stigma and encourage each other to disclose and evade structural barriers to access care. Nonetheless, most prisoner participants described a decline in such social networks due to lack of resources. The importance of social support for enhancing HIV care use in prisoners is well recognised [18, 20, 23, 24], suggesting a need for strengthening peer support programs in prison settings.

One prison-related factor appeared to facilitate HIV care use through various mechanisms. It assisted ILWH to re-assess and correct risky behavioural patterns that might have existed prior to their incarceration. Due to reduced access to their former social groups in which risky behaviour was entrenched, some ILWH achieved better medication adherence compared to other PLWH. Imprisonment also created a favourable environment for ILWH to learn from others about the negative health effects of risky behaviours such as discontinuation of medication. With a number of studies reporting improvements in ART adherence during incarceration in settings where there is a standard HIV care [49, 50, 60–63], these findings provide additional evidence on circumstances that may facilitate the effectiveness of comprehensive ART programs in prison settings.

Our study included prisoners who had been on ART for a relatively long period of time, but challenges of care use may vary depending on level of ART-related experience. Most barriers to, and facilitators of HIV care use identified in this study were consistently identified across the participating correctional facilities, suggesting the pervasiveness of the circumstances in Ethiopian prisons. However, larger quantitative studies are needed to draw conclusions that are representative of the prison populations. Further research is needed to more closely investigate the interconnections between patient privacy, disclosure and social stigma and their effect on HIV care use in a prison environment. Research is also required to investigate circumstances related to post-release HIV care.

## Conclusions

HIV care in the South Ethiopian prison system is likely to be improved if access to ART services is ensured. Access to standard care was substantially hindered by a lack of transport facilities, uncooperative security system, and poor collaborations between community and prison health care systems. Stigmatisation by fellow inmates and prison officers affected ILWH’s ability to disclose HIV status and led to despondency and a lack of commitment to use care. Insufficient supply of food in the prison system combined with a limited access to community nutritional programs aggravated medication side-effects, which in turn increased the chance of medication discontinuation. However, HIV care use was facilitated by ILWH’s: self-efficacy to disclose their HIV status and cope with the influences of social stigma, the presence of peer support, as well as vicarious experiences of adverse consequences of unhealthy behavioural patterns such as interrupting medication. Therefore, interventions that ensure: access to standard HIV care and health care provider support; preserve patient privacy and confidentiality, while promoting disclosure by reducing stigma; and enhance peer support and nutritional programs are strongly recommended.

## Data Availability

The datasets used and/or analysed during the current study are available from the corresponding author on reasonable request.

## Abbreviations

ART: Antiretroviral therapy
ILWH: Inmates living with HIV
PLWH: People living with HIV
SNNPR: Southern Nations, Nationalities and People’s Region
WHO: World Health Organization
